# Acute toxic kidney injury

**DOI:** 10.1080/0886022X.2019.1628780

**Published:** 2019-06-25

**Authors:** Nadezda Petejova, Arnost Martinek, Josef Zadrazil, Vladimir Teplan

**Affiliations:** a Department of Internal Medicine, University Hospital Ostrava, Ostrava, Czech Republic;; b Department of Clinical Studies, Faculty of Medicine, University of Ostrava, Ostrava, Czech Republic;; c Department of Internal Medicine III – Nephrology, Rheumatology and Endocrinology, University Hospital and Faculty of Medicine and Dentistry, Palacky University Olomouc, Olomouc, Czech Republic;; d Department of Nephrology, Institute for Postgraduate Education Prague, Vinohrady, Czech Republic

**Keywords:** acute kidney injury, toxic nephropathy, drug nephrotoxicity, plant nehrotoxicity, renal biomarkers, illicit drug nephrotoxicity

## Abstract

Substances toxic to the kidney are legion in the modern world. The sheer number and variety, their mutual interactions and, metabolism within the body are a challenge to research. Moreover, the kidney is especially prone to injury owing to its physiology. Acute kidney injury (AKI) induced by poisonous or primarily nephrotoxic substances, may be community acquired with ingestion or inhalation or nosocomial. Many nephrotoxic plants, animal poisons, medications, chemicals and illicit drugs can induce AKI by varying pathophysiological pathways. Moreover, the epidemiology of toxic AKI varies depending on country, regions within countries, socioeconomic status and health care facilities. In this review, we have selected nephrotoxic insults due to medication, plants, animal including snake venom toxicity, environmental, (agri)chemicals and also illicit drugs. We conclude with a section on diagnosis, clinical presentation and management of poisoning accompanied by various organ dysfunction and AKI.

## Introduction

An underlying feature in its pathophysiology is rapid decline in kidney excretory and metabolic functions with accumulation of nitrogen metabolism end-products such as blood urea and creatinine. A decrease in diuresis (oliguria/anuria) is often present. Patients with acute kidney injury (AKI) are classified into three clinical stages based on increase in creatinine and/or decrease in urine output, according to KDIGO (Kidney Disease Improving Global Outcomes) recommendations [1]. The etiology of AKI is generally divided, according to pathophysiological principle, into pre-renal, renal and post-renal. In recent years, there has been a change in definition based on epidemiology as: (1) community-acquired AKI, (2) nosocomial AKI and (3) AKI in the critically ill. AKI induced by poisonous or primarily nephrotoxic substances, may be regarded as community acquired with ingestion or inhalation of toxic substances or nosocomial, i.e. hospital acquired. In special circumstances, the AKI can develop in critically ill patients in intensive care units (ICUs) after administration of nephrotoxic treatments or, in susceptible patients undergoing radiological examination with the use of high osmolar iodinated radiographic contrast media [2].

## Search strategy and selection criteria

PubMed, MEDLINE, EBSCO, UptoDate and Cochrane databases were searched using the following terms and their abbreviation: “toxic nephropathy”, “toxic acute kidney injury” , “drug nephrotoxicity”, “plant nephrotoxicity”, “illicit drug toxicity”, “microRNA”, “biomarkers in acute kidney injury”. Publications were identified according to the authors best knowledge of the issue. Human and experimental trials, case series and reports were considered in the literature search. All data were abstracted systematically from June 2018. Non-English publications and literature sources were also included and carefully translated. The reference lists of published works were also searched for further relevant papers.

## Epidemiology and etiology

The causes of acute toxic kidney injury have changed substantially over the centuries. In Medieval Europe, for example, poison became a popular form of killing, due to the public availability of apothecaries and this appeared to be a main cause of renal and other toxicity. In current times, according to one retrospective, case-control study using the data of the American Association of Poison Control Centers’ National Poison Data System (NPDS), of the approximate 16.8 million exposures reported to the NPDS, there were 16 444 single substance exposures with renal effects, of which 9074 cases had serious kidney complications *55.2%). The forms of presentation of acute renal complications were, increased creatinine, and/or oliguria/anuria, and/or renal failure. Poisoned patients with renal impairment had higher rates of renal replacement therapy initiation (27.7%) and death (10.9%) [3]. According to 34th annual report of NPDS, human exposures to toxic substances with less serious outcome have decreased 2.59% per year during the last decade while those with more serious organs damage and outcome have increased by 4.39% per year in last two decades [4]. The etiology of community acquired AKI greatly depends on geography and varies between regions. One Indian study on 2405 patients describing the changing epidemiology of community acquired AKI over 26 years, revealed increase in an overall incidence of AKI of 1.95 (first 13 years) and 4.14 (second 13 years) per 1000 admissions. Other causes aside, the incidence of nephrotoxic AKI due to antibiotics had since declined while new causal agents have emerged, including nonsteroidal anti-inflammatory drugs (NSAIDs), angiotensin-converting enzyme inhibitors (ACEi), chemotherapeutic and antiviral medications [5]. In one large Chinese multicenter retrospective cohort study of 659 945 hospitalized adults from a wide range of clinical settings, the incidence of community acquired AKI and hospital acquired AKI was 2.5% and 9.1%, respectively. More than 40% of AKI cases were possibly drug-induced and it is remarkable that traditional Chinese medicine remedies were the most common drug exposure before the occurrence of AKI (community acquired 15.3% and hospital acquired 16.2%) [6]. Traditional herbal medicine is prevalent in most countries in Asia but the supervision of pharmacology is not sufficiently managed and the medicines are usually prescribed by practitioners of traditional medicine with lack of medical facilities and accurate knowledge of the adverse effects [7]. Thus, the epidemiology of AKI varies depending on country, regions within countries, socioeconomic status and health care facilities. There are interesting differences between East and West and between low-income and high-income countries. The lower incidence of AKI in Asian regions may be partly due to less recognition. Overall however, AKI patients in South Asia are significantly younger than in East Asia and Western countries with less known comorbidities. In addition to traditional herbal medicine toxicity, there are environmental toxins such as snake, wasp and bee venoms and the influence of severe tropical diseases, e.g., malaria, hemorrhagic fever, dysentery and leptospirosis [8].

## Medication-induced nephrotoxicity

**Figure 1.**
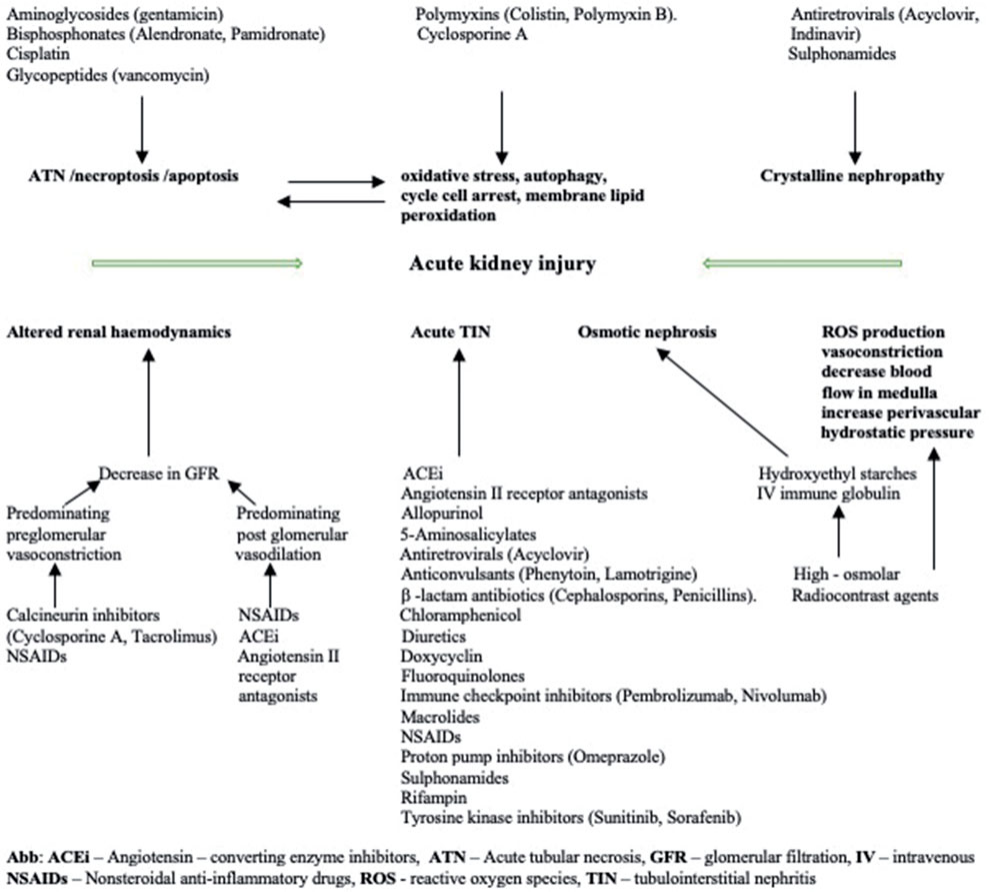
Drug-induced nephrotoxicity – cellular mechanisms [12–23].

There is a steady, exponential increase in medication-induced nephrotoxicity with special emphasis on antimicrobial therapy at a time of increasing pharmacological resistance in the case of severe bacterial infections. Many bacterial species produce effective enzymes necessary for self-protection but this leads to intractable resistance and progression of critical conditions with septic AKI development. These aside, many antibiotics, immunosuppressants and analgesic agents have considerable nephrotoxicity via different pathophysiological mechanisms. Known adverse drug reactions are generally divided into two groups: (1) unpredictable, immunologically mediated and dose-independent representing 15–20% of all reactions (e.g., β-lactams, penicillins) and (2) predictable and dose-dependent comprising up to 80% (e.g., vancomycin, gentamicin, colistin) [9]. The first type of reaction is usually described as hypersensitivity or drug allergy which can range from mild forms to severe life-threatening anaphylactic events. The prevalence of drug allergies documented for two decades in electronic health records is, an average 1.95 events per patient with an incidence of 35.5% in a study population of 1 766 328 patients. The most common drug allergies were to antibiotics namely penicillins (12.8%) and sulfonamide (7.4%). The others were to opiates (6.8%) and NSAIDs (3.5%). Noteworthy is the fact that females and white patients were more prone to adverse reactions from common medications, except from, e.g., NSAIDs or ACEi [10]. Acute tubulointerstitial nephritis (ATIN) with varying stages of renal injury (decrease in glomerular filtration), eosinophilia, hematuria, mild proteinuria (<1.0 g/day), allergic skin rash and fever after drug exposure are one of the typical clinicopathological presentations of immunological drug reaction. In a single center Chinese prospective cohort study including patients with biopsy proven ATIN of any etiology, drug-induced ATIN was diagnosed in 50% of patients. The other etiological factors were autoimmune related disease and TIN with uveitis. In addition, from multivariable analysis, independent risk factors for poor long-term renal outcome were female sex, older age, hypertension and recurrent renal failure [11]. The pharmacological groups of drugs associated with ATIN are very broad and include e.g. antibiotics (β – lactams, chloramphenicol, doxycyclin, rifampin, sulfonamides, fluoroquinolones, macrolides), diuretics, antihypertensive agents (ACEi, angiotensin II receptor antagonists), NSAIDs, proton pump inhibitors, immune checkpoint inhibitors, antiretroviral agents and tyrosine kinase inhibitors (Figure 1) [12].

### Cyclosporine A nephrotoxicity

A recent toxicological report included changes in described cyclosporine A (CyA) nephrotoxicity with effects on renal haemodynamics. CyA can induce autophagy of renal tubular cells with oxidative and endoplasmic reticulum stress, increase in mitochondrial reactive oxygen species production and activation of a variety of signaling pathways leading to membrane lipid peroxidation. The dose-related and predictable adverse reaction can induce severe damage of renal parenchyma with pathological presentation as acute tubular necrosis, apoptosis, oxidative stress or autophagy [24].

In current intensive care practice, some broad spectrum antibiotics with known nephrotoxicity are used. These need therapeutic drug monitoring to prevent toxic serum concentrations while maintaining therapeutic effect.

### Vancomycin nephrotoxicity

The glycopeptide antibiotic agent, vancomycin is a very potent bactericidal time-dependent antimicrobial agent for treating methicillin-resistant *Staphylococcus aureus* used in intravenous form. In the last decade, the best pharmacokinetic/pharmacodynamic parameter that correlates with vancomycin efficacy is the ratio of the 24 h area under the curve to the minimum inhibitory concentration (AUC/MIC) > 400. Vancomycin related nephrotoxicity can vary from mild to severe AKI necessitating renal replacement therapy (RRT) initiation [25]. The vancomycin nephrotoxicity may depend on concomitant nephrotoxic treatment, advanced age, a steady-state vancomycin trough concentration of 15 μg/mL or more with a reported incidence of 8.1% for vancomycin alone and 16.3% for concomitant piperacillin – tazobactam therapy [26]. Experimental *in vitro* studies have shown the underlying pathophysiological principles of vancomycin nephrotoxicity of proximal tubular cells is based on induction of depolarization of the mitochondrial membrane with production of mitochondrial reactive oxygen species (ROS) and peroxidation of mitochondrial phospholipid cardiolipin. The activation of caspase-9 and caspase-3/7 is accompanied by vancomycin-induced apoptotic cell death [27,28].

### Aminoglycoside nephrotoxicity

Aminoglycoside antibiotics are broad spectrum concentration dependent antimicrobials with effectiveness against gram-negative bacteria and in synergy with β – lactams antibiotics are active against *Staphylococcus* and *Enterococcus*. The reported incidence of aminoglycoside-induced AKI is approximately 33% depending on the presence of shock in critically ill elderly patients and therapy duration [29]. The newer facts about gentamicin-induced nephrotoxicity show four routes toxicity: (1) direct tubular, (2) glomerular, (3) vascular toxicity and (4) induction of oxidative stress with production of ROS in mitochondria. Gentamicin is eliminated mainly by glomerular filtration and small amounts are actively reabsorbed and accumulate in proximal tubular cells, leading to interaction with cell membranes and organelles. The accumulation of gentamicin in lysosomes causes phospholipidosis with resulting cell necrosis. Gentamicin can decrease glomerular filtration due to mesangial contraction, mesangial cell proliferation and decrease in renal blood flow due to increase in vascular resistance [30,31].

### Colistin nephrotoxicity

Colistin is a polypeptide polymyxine antibiotic agent with well-known nephrotoxicity. There is a revival of interest in it, however due to its effectiveness against nosocomial multidrug resistant bacterial groups such as *Pseudomonas aeruginosa* and *Acinetobacter baumanii*. The reported incidence of colistin-induced nephrotoxicity in critically ill patients in ICUs is 54.2% with higher rates in patients with malignancy and after abdominal surgery [32]. Colistin causes acute tubular necrosis due to tubular epithelial cell membrane deterioration and influx of cations, anions and water, leading to cell swelling and lysis [33].

However, the cellular mechanisms of polymyxin and colistin nephrotoxicity include oxidative stress, apoptosis (via mitochondrial, death receptor, and endoplasmic reticulum pathways), cell cycle arrest, and autophagy [13].

### Cisplatin nephrotoxicity

Cisplatin is a chemotherapeutic agent commonly used for treatment of various type of malignancies. The reported risk ratio for development of cisplatin nephrotoxicity in comparison to other treatments is 1.75, according to a meta-analysis of 2359 human studies [20]. The pathophysiological and biochemical process of cisplatin nephrotoxicity is very complex and includes the drug accumulation in the tubular epithelial cells, activation of apoptosis via death receptors like TNF-α and Fas in the plasma membrane, activation of endoplasmic reticulum stress or by cycle cell arrest [21].

### Iodine contrast nephrotoxicity

Contrast – medium induced nephropathy defined as iatrogenic disease occurs after intravascular injection of high osmolar iodinated radiographic media with increase in serum creatinine >25% or a 0.5 mg/dL (44 µmol/L) absolute increase over baseline at least 24–72 h after the contrast exposure. Patients who receive iodinated contrast – medium are approximately at 5.5% risk for AKI development [22], which highly depends on other comorbidities, status of hydration and renal functions before examination. The pathophysiology of contrast – medium induced nephropathy is presumed to be a combination of factors: (1) direct toxicity to the renal tubular cells, (2) production of reactive oxygen species, (3) low blood flow in the medulla, (4) increased perivascular hydrostatic pressure or (5) changes in concentration of some vasoactive substances (e.g., nitric oxide, adenosine) [23].

## Plant nephrotoxicity

Nature has always been a prolific source of information and treatment options for humans from ancient times. Plants and their extracts have been used in medicine for centuries for their curative effects in a large number of pathological circumstances. Many of our primary medicines are of plant origin, e.g., digoxin manufactured from *Digitalis lanata,* morphine and codeine from *Papaver somniferum*, chemotherapeutic agents Paklitaxel – an alkaloid from *Taxus brevifolia*, vinca-alkaloids (vinblastin, vincristin) from the periwinkle plant *Catharanthus roseus*, the important hepatoprotective agent silymarin extracted from *Silybum marianum* or milk thistle and quinine from *Cinchona pubescens* [34–36].

### Chinese herbal medicine-induced nephrotoxicity

In some, mostly developing countries around the world, traditional herbal medicine is one of the most important aspects of illness prevention, cure and treatment. Some have a thousand-year history such as Chinese and Persian herbal medicine. In recent times, herbal medicine has become very popular in developed countries mainly Western Europe and the United States as alternative medicine. However, some pharmacological ingredients in Chinese herbal medicine have been identified as having an influence on AKI, e.g., aristocholic acid, the flavonoids – sciadopytisin, oxidative degradation products, andrographolide, active moiety triptolide, glycyrrhetinic acid, ephedrine and colchicine [37]. Aristocholic acid is a toxic compound of *Aristocholia* sp. and is associated with progressive chronic tubulointerstitial fibrosis with rapid loss of renal function. However, acute aristocholic nephropathy can superimpose on primary chronic glomerulonephritis with a presentation of severe proteinuria [38]. The information on flavonoids and their effects on renal function and parenchyma appears to be controversial. According to some resources, flavonoids are renoprotective owing to their multiple beneficial properties, e.g., antioxidation, anti-inflammatory, blood pressure reduction and increase in nitric oxide, which counteract the deleterious effects of kidney injury that can lead to chronic kidney disease. Various types of flavonoids also show anticancer activity toward renal cancer cells, inducing cell cycle arrest, apoptosis and suppression of cancer cell proliferation [39]. Nevertheless, several human case reports showed acute renal failure induced by sciadopitysin – a compound of *Chinese yew (Taxus celebica)*, a type of flavonoid. Injured patients initially presented with fever and gastrointestinal disorders after ingestion of a single large dose or long-term small doses, and over 2–9 weeks symptoms worsened with hemolysis, cholestatic hepatitis and disseminated intravascular coagulopathy. Renal biopsy showed acute interstitial nephritis with acute tubular necrosis [40]. Andrographolide a diterpenoid lactone, the main component of the traditional medicinal herb, *Andrographis paniculata* – an herbaceous plant of the family Acanthaceae, is widely used in Chinese, Indian or Asian medicine in oral, intra-muscular or intravenous form for the treatment of upper and lower respiratory tract infection, e.g., cold, pneumonia, tonsillitis or in the case of bacillary dysentery. *A. paniculata* produces large quantities of the anti-inflammatory, diterpenoid lactones andrographolide, neoandrographolide and their analogs and genes encoding diterpenoid synthases have been recently discovered [41]. It is very desirable to draw attention to the positive properties of the drug, published in recent scientific reports highlighting its anticancer, anti-angiogenic (via VEGF pathway), anti-inflammatory and anti-viral activity [42–44]. However, retrospective analysis of patients treated by intravenous forms of andrographolide, revealed some cases of AKI which may occur shortly after infusion with clinical signs of flank pain, decreased urine output and gastrointestinal symptoms with nausea and vomiting. Kidney biopsy in two patients showed pathological changes associated with acute tubular necrosis [45]. For this reason, it is necessary to monitor renal function during the drug administration and to be careful in case of chronic kidney disease in a patient’s history.

### Persian plants nephrotoxicity

Besides Chinese, Indian and African herbal medicine, the traditional herbal remedies of Persia show the deep historic relationship between religion, culture, plants and human health. The use of herbal medicines for treating renal conditions such as inflammation and urolithiasis and, some aspects of toxicology is described in the *Hidayat* in 1058 A.D. [46]. Over the centuries, the therapeutic approaches changed and many potential nephrotoxic, also renoprotective plants have been identified, e.g., *Amaranthus* spp., *Artemisia absinthium, Rumex* spp., *Pennisetum* spp., *Portulaca oleracea* and *Cymbopogon* spp. [47]. The published literature and scientific information on the nephrotoxic effects of *Amaranthus* sp. remain controversial. Some spp. are probably able to superimpose the toxic effects of plants producing oxalates which cause precipitation of calcium as calcium oxalate crystals in a number of tissues including the renal tubules and this can induce renal tubular necrosis. In one Australian report, a large number of animals (lambs) died after *A. hybridus* and *A. retroflexus* ingestion. The postmortem analysis showed bilaterally swollen kidneys with diffuse pale cortices strongly suggestive of acute toxic nephrosis [48]. The potentially valuable properties of *Amaranthus* greatly depend on its preparation before using. And in some countries in Africa (Uganda, Nigeria) and Iran (Persia), *A. hybridus* is used for its antioxidant and anticancer effects. It contains appreciable amounts of proteins, fat, fiber, carbohydrate and has calorific value, amino acids, minerals, vitamins, and generally a few of toxicants [49–51]. Renoprotective effects have been observed for *Cymbopogon citratus* (Gramineae) known as lemongrass, in animal studies with rabbits and the aminoglycosides induced renal injury. Animals treated with *C. citratus* histologically exhibited intact parenchyma with no evidence of tubular necrosis or any significant abnormality in glomeruli [52].

### African herbal medicine induced nephrotoxicity

**Figure 2.**
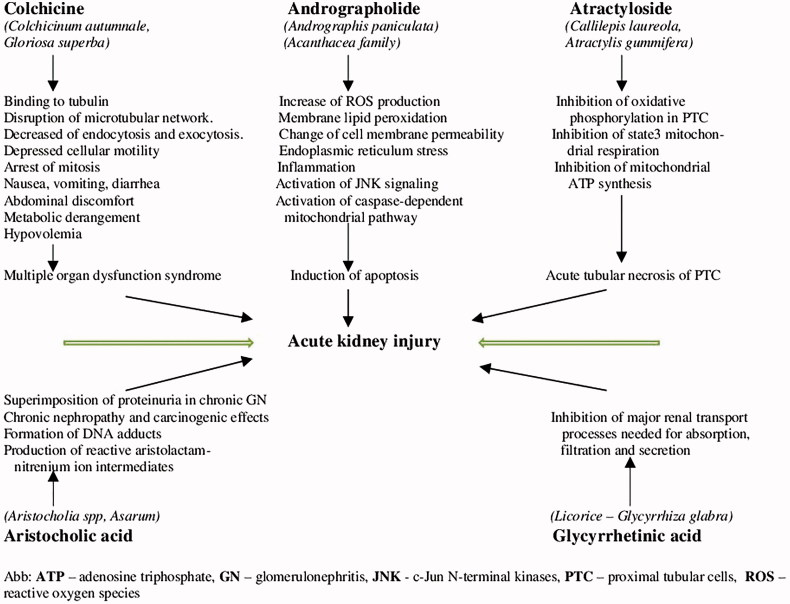
Pathophysiology of plants-induced nephrotoxicity [37,38,53–58].

In addition to these historically authenticated herbal medicines, in South Africa, the Democratic Republic of Congo, Zimbabwe, and Zambia common nephrotoxic plants are *Impila* meaning health in Zulu (*Callilepis laureola*) and *Euphorbia matabalensis* with acute tubular necrosis as a result of direct renal injury or ischemia due to dehydration (Figure 2) [59].

According to recent scientific research on animals, the main nephrotoxic component in *Euphorbiacae* sp. is petroleum ether. Metabolomic evaluation of *Euphorbia pekinensis* (known also in China) showed a disturbed metabolic pathway in the renal metabolic profiling. The most affected were purine, aminoacids, phospholipids and sphingolipids metabolic pathways [60].

Indications for the use of *Impila* are very broad, from wording off evil spirits, to treating disease problems such as gastritis, pneumonia and tape worm infestations, to facilitating labor in pregnant women. General symptoms of *Impila* intoxication include vomiting, diarrhea, abdominal pain, neurological signs such as disturbance in consciousness and convulsions, and acute liver and renal failure with typical clinical and laboratory presentation [57].

The mechanism by which *C. laureola* and thistle *Atractylis gummifera* induce nephrotoxicity is not completely known. However, the basic nephrotoxin in these herbal remedies has been identified as a diterpene glycoside – atractyloside, which in animal studies has been found to affect renal cortical mitochondria and proximal tubular fragments. There is evidence that the nephrotoxicity may be caused by altered mitochondrial respiration and inhibition of oxygen uptake by the intact cells [58].

However, a plethora of plants growing in Europe are referred to as very toxic but also an invaluable and indispensable source of medicinal substances for the pharmaceutical industry as has been mentioned above.

## Animal venom nephrotoxicity

One of the most serious complications worldwide of poisonous snake bites mainly due to the families Viperidae, Colubridae, Elapidae, Hydrophiidae and Atractaspididae is acute renal failure. Snake venoms contain a large amount of active toxins which can induce AKI via several pathological pathways [61,62]. For example, in Japanese Islands, *Protobothrops flavoviridis*, belonging to the Viperidae family, is one of the major poisonous snake in terms of its toxicity and hemotoxicity and the damage its bite causes. In pathophysiological terms, snakebite AKI may be a consequence of its hemotoxic effects leading to severe rhabdomyolysis [62]. Severe congestion, petechial hemorrhage in various tissues and acute tubular necrosis of proximal tubular cells with intact basement membrane have been observed in mice envenomated by hump-nosed pit vipers *Hypnale* from the Viperidae [63]. *Hypnale* is a very venomous snake found in Sri Lanka and India. In a prospective human observation study of 465 hump-nose viper bitten patients, 9.5% developed AKI. Hematuria, microangiopathic hemolysis, oliguria, thrombotic thrombocytopenic purpura and hemolytic uremic syndrome have been observed in clinical presentation of envenomation and 14% of AKI patients died due to renal complications and coagulopathy [64].

Hymenoptera more specifically, bees and wasps are widespread throughout the world and their stings can be only harmless with erythematous and edematous local reaction or deadly due to Ig-E mediated anaphylactic shock and following cardiovascular or multiple organ complications. A hypersensitivity reaction can lead to rhabdomyolysis with skeletal muscle cell damage and consequent myoglobinuria and AKI. Another pathophysiological mechanism is based on activation of disseminated intravascular coagulation with tubulointerstitial and acute tubular cell injury [65].

## Environmental (Agri)chemical nephrotoxicity

**Figure 3.**
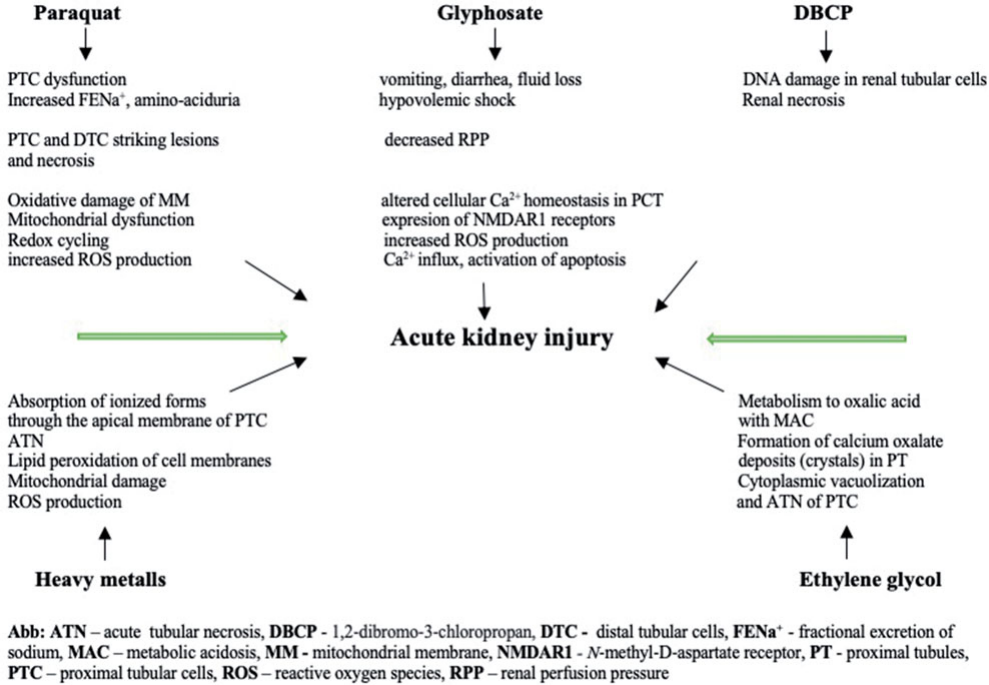
The mechanisms of selected environmental and (agri)chemical nephrotoxicity [69–82].

The fatal, rapidly progressive chronic kidney disease and unremitting epidemic of inexplicable acute failure of renal function in agricultural workers and communities, especially in sugar canes cutters is known as Mesoamerican nephropathy. A some research has been done on chemicals, heavy metals and also on potential infection etiology. The disease is common in countries of Central America: Nicaragua, Guatemala, El Salvador, Costa Rica and Honduras. Due to the estimated number of deaths of 20 000 to 2010 [66], the Pan American Health Organization at the 52nd Directing Council in 2013 adopted a fundamental position on disease control, surveillance and follow-up. According to the results, the disease is most common in underprivileged young men and workers on farms with an extensive health, social and economic burden on affected countries. The causes appear to be multifactorial, including environmental toxins (e.g., agrichemicals) in combination with the occupational risks of high temperatures and inadequate rehydration with water intake [67]. The acute form of the disease presents with gastrointestinal disorders (nausea and vomiting), arthralgia, headache, myalgia, weakness, back pain, neurological symptoms as paresthesia and tingling sensation. An aspect of the condition has been linked by some authors to infection etiology, e.g., leptospirosis and hantavirosis as neglected tropical diseases but this is not a single causative factor [68]. The pathophysiological mechanisms of selected environmental and (agri)chemical nephrotoxicity are presented in Figure 3.

However, the uncontrolled and dangerous use of agrichemicals as pesticides in sugar cane production is a serious problem in developing countries. Some organophosphates have been identified as nephrotoxic such as compounds containing glyphosate – (phosphonomethyl) glycine. Organophoshates are most frequently used as herbicides, pesticides and unfortunately in chemical warfare predominantly as neurotoxic agents. The toxicity of organophosphates is generally based on the irreversible inhibition of acetylcholinesterase which in physiological condition brakes down acetylcholine. Other toxic chemical substances are 1,1′-dimethyl-4,4′ bipyridinium dichloride – Paraquat and 1,2-dibromo-3-chloropropane and 2,4-dichlorophenoxyacetic acid [71].

### Glyphosate nephrotoxicity

Glyphosate (phosphonomethyl) glycine, is an active ingredient in glyphosate-surfactant herbicides used worldwide especially in Asia and the countries of Mesoamerican region. Under *in vitro* conditions is a weak inhibitor of acetycholinesterase as has been shown in animals [72]. High systemic concentration of glyphosate would be expected in ingestion or self-poisoning. Acute self-poisoning after intentional or accidental oral ingestion can be lethal and most severely poisoned patients die from acute renal failure accompanied by other organ complications such as acute respiratory distress, neurological and severe gastrointestinal symptoms with excessive loss of fluids due to vomiting and diarrhea resulting in hypovolemic shock [73]. The histopathological analysis of renal injury in glyphosate poisoned animals has revealed significant necrotic and apoptotic damage to proximal tubular cells and thickened ascending limb of the loop of Henlé in the outer medulla and cortex [74].

### Paraquat nephrotoxicity

The nephrotoxicity of the potent herbicide, paraquat (1,1′-dimethyl-4,4′ bipyridinium dichloride), following ingestion has been known for more than 30 years when its effect on renal function was first described. Proximal tubular cell dysfunction with increased fractional excretion of sodium, phosphorus and uric acid as well as marked amino-aciduria and glycosuria has been documented in fatal case reports [75]. The histopathological findings from animal studies with paraquat poisoning are dose-dependent, with striking proximal and distal tubular cell lesions showing coagulation necrosis with tubular cell loss [76]. The main organs injured by paraquat are kidney and lung and pathophysiological mechanism of paraquat toxicity has been established on its redox cycling with production of reactive oxygen species and oxidative stress injury [77].

### 1,2-Dibromo-3-chloropropane nephrotoxicity

1,2-dibromo-3-chloropropane (DBCP) is a halogenated aliphatic hydrocarbon widely used in agriculture as a pesticide – fumigant with nematocidal activity. DBCP is reasonably anticipated to be a human carcinogen based on sufficient medical evidence of carcinogenicity from experimental animal studies. Oral exposure to 1,2-dibromo-3-chloropropane caused tumors at several different tissue sites [78]. Experimental extensive acute tubular necrosis has been observed in various animal species following intraperitoneal injection of DBCP [78].

### Heavy metals nephrotoxicity

Heavy metals (cadmium, uranium, lead, mercury) are environmental and industrial toxins which have long been recognized for their potential carcinogenicity to humans and animals after ingestion or any type of acute or chronic exposure. The clinicopathological presentation and renal injury differ substantially for acute and chronic intoxication. Ionized forms are found in acute poisoning. The difference between acute and chronic intoxication could result from the concentration, the nature and form (free and bound) of the toxic heavy metals. More than 90% of filtered ionized heavy metals are absorbed through the apical membrane of the proximal tubular cells leading to acute tubular necrosis in acute intoxication [80–83]. In the case of chronic ingestion, the nephrotoxicity of heavy metals is mainly due to the transport of the filtered conjugate form on the thiol groups of specific or nonspecific proteins, whereas in acute poisoning the free ionized form and conjugates with short peptides and thiol-amino acids can directly induce renal injury [80].

### Toxic alcohols nephrotoxicity

Acute poisoning with the toxic alcohol ethylene glycol, may be accidental or intentional with typically high anion gap metabolic acidosis and accompanied by specific clinical symptoms including neurological, cardiopulmonary and renal phases. Ethylene glycol intoxication is associated with calcium oxalate deposits in various tissues and causes severe AKI with histological findings of acute tubular necrosis, cytoplasmic vacuolization and refractile calcium oxalate crystals in proximal tubules [84]. In contrast to other toxic alcohols (ethylene glycol and methanol), intoxication with potentially lethal isopropyl alcohol is accompanied by normal acid-base balance, anion gap and pH, but is associated with high osmolar gap. The reason for this lies in the liver metabolism of isopropyl alcohol by alcohol-dehydrogenase to acetone and none of its metabolites are organic acids. Moreover, acetone can cause laboratory assay interference in the determination of serum creatinine concentration giving a pseudorenal failure laboratory picture [85].

## Illicit drug abuse nephrotoxicity

Illicit drug use is a global problem with multiple impact on health care systems, morbidity, mortality, long-term psychiatric care and economies. In many countries, military counter narcotics campaigns are being conducted to destroy drug cartels and producers. The most significant types of illicit drugs associated with acute renal damage in abusers are synthetic cannabinoids (sCBs), amphetamines, cocaine and heroin.

### Synthetic cannabinoids nephrotoxicity

Synthetic CBs known as, spice, are recreational drugs with psychoactive effect which bind to the same, type 1 (CB1R) and type 2 (CB2R) receptors as endogenous cannabinoids – lipid ligands (e.g., anandamide and 2-arachidonoylglycerol) and Δ9-tetra-hydrocannabinol (THC) the psychoactive constituent of cannabis. The sCBs were originally developed to clarify the principle of cannabinoid biochemistry but due to their considerable psychoactive effects, they rapidly emerged on the drug market as cheaper and more efficient alternatives to cannabis [86]. However, their effects are unpredictable and they are more toxic than cannabis. CBR1 receptors are located in multiple sites of the central and peripheral nervous system with effects on food intake and energy expenditure. However, both receptor types are found and distributed to some extent in different renal cells including proximal tubular cells, arterioles, collecting ducts, podocytes, mesangial cells and glomeruli [87]. The role of renal endocannabinoid receptors has been intensively studied, especially in association with diabetic nephropathy and obesity-related kidney disease. CB1R antagonism and CB2R agonism reduce albuminuria and, to some extent, renal fibrosis in both type of diseases [88,89]. When we are talking about acute kidney damage, the most important is sCBs effect on renal hemodynamics which is likely involved in regulating renal blood flow and may play a role in tubular sodium reabsorption [86,90]. The clinical presentation of sCBs toxicity includes altered mental status with hallucinations, delusions, irritability, confusion, seizures, agitation or lethargy, gastrointestinal symptoms as nausea, vomiting with excessive dehydration, tachycardia and hypertension [91].

Renal biopsy on people with sCBs poisoning and acute renal failure, revealed acute tubular necrosis, proximal tubular dilation with epithelial cell vacuolization and apical blebbing, areas of denuded tubular basement membranes indicating focal epithelial cell loss [92,93]. The exact pathophysiological mechanism of sCBs induced AKI is unknown but one important mechanism is considered to be decreased renal perfusion pressure due to hypovolemia and dehydration caused by excessive vomiting. However, recent scientific data from Portugal, in an *in vitro* study on human proximal tubular cells, demonstrated a possible mechanism of sCBs nephrotoxicity based on impaired endocannabinoid-mediated regulation of mitochondrial function homeostasis and triggering of apoptosis. The synthetic cannabinoid used in the study, XLR-11 – (1-(5-fluoropentyl) -1*H*-indol-3-yl) (2,2,3,3-tetramethyl-cyclopropyl) methanone – in principle, induced hyperpolarization of the mitochondrial membrane, increased ATP production and promoted Bax translocation from the cytosol into mitochondria. These phenomena were followed by an initiation of caspase-3 activity and chromatin condensation, which indicated activation of apoptotic cell death pathways [94]. When acute tubular necrosis was confirmed on kidney biopsy, more other pathophysiological pathways must be involved in the whole mechanism of sCBs induced renal damage.

### Cocaine nephrotoxicity

Cocaine (benzoylmethylecgonine) is biochemically a crystalline tropane alkaloid, extracted from the leaves of the *Erythroxylon coca* plant from the western parts of South America. The use of cocaine as a recreational drug is predominantly based on its strong psychostimulatory effects with increased feelings of well-being and euphoria and also decreased need for food and sleep. It stimulates the sympathetic nervous system with vasoconstriction, oxidative stress, endothelial dysfunction and change in prostaglandin concentration. These pathophysiological mechanisms can lead to adverse manifestation such as myocardial infarction, acute ischemic stroke or sudden death.

The clinical presentation of acute cocaine toxicity usually includes tachycardia, psychological disturbances as hallucinations, agitation and aggressive behavior, mydriasis, hyperreflexia, hyperpyrexia, acid–base disturbance and arterial dissection [95]. Cocaine induced AKI is a result of a number of pathological processes and affected pathways, e.g., (1) rhabdomyolysis with disruption of skeletal muscle cells due to ischemia and vasoconstriction, (2) acute interstitial nephritis, (3) renal infarction due to vasoconstriction and thrombosis, (4) thrombotic microangiopathy and malignant hypertension due to endothelial injury with thrombocyte activation [96].

Kidney biopsy confirmed acute tubular interstitial impairment with inflammatory infiltration and edema [97]. However, at the cellular and molecular level, the situation is more complex and apart from toxicity to other organs, cocaine and its metabolites, mainly norcocaine showed direct cytotoxic effects on human proximal tubular epithelial cells in one *in vitro* study [98]. Depending on cocaine and norcocaine concentration, intracellular glutathione and ATP levels decreased with following inhibition of mitochondrial activity and impaired glutathione redox cycle. Activation of apoptosis was the observed result of both pathophysiological processes [98].

### Heroin nephrotoxicity

Heroin (diacetylmorphine) a derivate of morphine an alkaloid from the opium poppy plant *Papaver somniferum,* is usually injected intravenously in drug addicts. It causes severe psychological and physical dependence and leads to complete degradation of the drug users’ personality. At the current time, substance abuse is one of the most pressing social crises affecting many countries, and it impacts the economic and cultural aspects of communities [99]. Heroin induced nephropathy is mostly associated with concomitant chronic hepatitis C and human immunodeficiency virus infection with secondary focal segmental glomerulosclerosis or membranoproliferative and membranous glomerulonephritis with a clinical presentation of nephrotic syndrome. One case report of a biopsy proven heroin crystal nephropathy demonstrated on electron microscopy, intratubular crystals with peripheral radiating spicules, located within tubular epithelial cells and in the tubular lumen, in a patient with clinical presentation of severe AKI [100].

A German postmortem analysis of 129 illicit drug abusers revealed a broad and nonspecific spectrum of pathologic alterations of renal parenchyma, such as glomerular obliteration, interstitial inflammation, calcifications, interstitial fibrosis and tubular atrophy [101].

### Amphetamines nephrotoxicity

Amphetamines are synthetic, widely abused psychoactive drugs with significant stimulant, euphoric, anorectic, empathogenic, entactogenic and hallucinogenic properties. Common in clinical psychiatric practice are amphetamine, methamphetamine and 3,4 methylenedioxymethamphetamine (MDMA, Ecstasy). However, in human history some natural amphetamines have been used for centuries through the ingestion or chewing the leaves of the *Catha edulis* plant (Khat) in East Africa and Arabia or plants of the genus *Ephedra e.g. Ephedra sinica.* The biochemical structure of amphetamines is similar to the monoamine neurotransmitters with competitive action at membrane transporters of dopamine, norepinephrine and serotonin. The results of competitive membrane action are blocking of reuptake and induction of the reverse transport of endogenous neurotransmitters [102]. The clinical presentation of MDMA poisoning varies from hyperpyrexia, arrhythmias, serotonin syndrome, neurological symptoms with seizures, sudden death or coma. In vitro study using rat and human proximal tubular cells showed no direct nephrotoxicity of MDMA or methylenedioxyamphetamine (MDA) in varied concentrations, but alpha-methyldopamine (alpha-MeDA) itself and conjugation with glutathione (GSH) to 5-(glutathione-S-yl)-alpha-MeDA were extremely potent nephrotoxicants with approximately 70–80% cell death. Thus, the metabolism of ecstasy on the apical membrane of renal proximal tubular cells with the extracellular event of redox cycling appears to be the possible part of pathophysiological pathway of MDMA nephrotoxicity [103].

**Figure 4.**
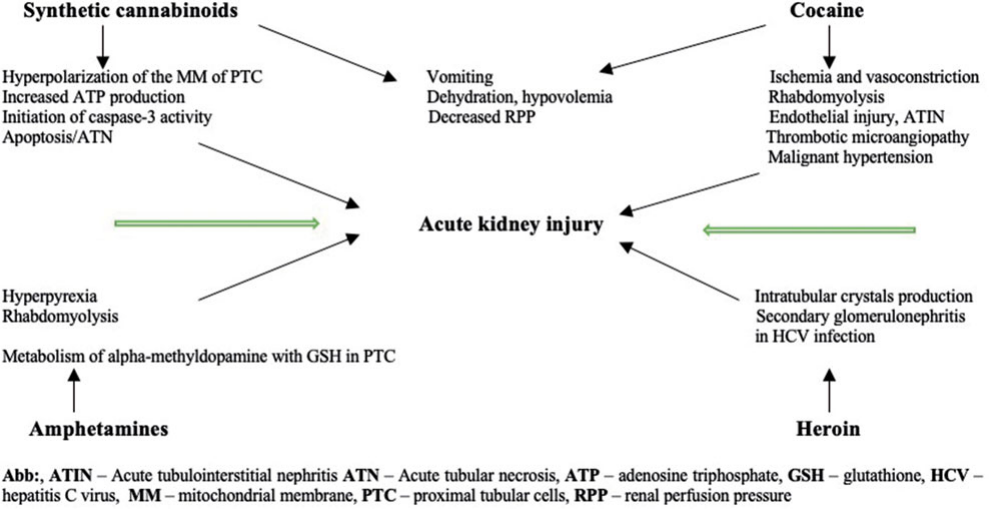
Mechanisms of illicit drug nephrotoxicity [92–104].

The summary of pathophysiological mechanisms involved in illicit drugs nephrotoxicity is presented in Figure 4.

## Clinical presentation

The clinical picture of acute intoxication accompanied by AKI usually depends on pharmacokinetics/pharmacodynamics properties of the toxic substance and on the severity of renal impairment. In general, venomous substances can affect the human organism via different routes: by oral ingestion, inhalation and through the skin. Beside the renal damage and failure, other organs and systems can be affected with clinical presentation of gastrointestinal symptoms (nausea, vomiting, diarrhea, salivation) with dehydration and hypotension due to volume loss, neurotoxic signs with crumps, unconsciousness, dizziness, seizures, coma or hepatotoxic signs with jaundice or cardiac and respiratory failure. The first step in these situations besides resuscitation of vital functions with nonspecific therapy is patient history with diagnosis of intoxication and exact knowledge of the availability of any antidote. Nonspecific therapy in any situation of suspected or confirmed poisoning includes vital function resuscitation (airway, breathing, circulation), introduction of venous access, gastric tube and urine catheter insertion and tracheal intubation in patients with acute respiratory failure [12,105]. Absolutely essential is toxicological examination of blood, urine and stomach fluid if possible. Bolus of intravenous crystalloids solution, gastric lavage and activated charcoal administration 25 g every 4 h are an intrinsic part of treatment in every case of intoxication. Blood results can reveal severe metabolic derangement, especially in ions or acid-base disturbances. The clinical presentations of selected nephrotoxic substances with required antidotes and other therapeutic options are summarized in Table 1.

**Table 1.** The clinical presentation and treatment options of selected nephrotoxic substances.

Type of toxin/poisonous substance	Clinical/laboratory presentation	Specific antidote and treatment options
Ethylene glycol [106–109]	Alteration of consciousness, hypotonia, hyporeflexia, seizures, ataxia, coma, heart failure, ARDS, oliguria, flank pain, AKI–ATN, tachycardia, severe MAC, urinary oxalate presents	10% Ethanol IV – 7.5 mL/kg LD and 1–2 mL/kg/h in nondrinkers and 2–4 mL/kg/h in drinkers to achieve a blood ethanol concentration of 100 mg/dL (22 mmol/L) Fomepizole IV – 15 mg/kg LD and 10 mg/kg every 12 h and after 48 h 15 mg/kg every 12 h Acute IHD in severe MAC, AKI and EG levels > 50 mg/dL
Isopropanol [106,110]	Nausea, vomiting, abdominal pain, gastritis, hematemesis, headache, dizziness, confusion, stupor, coma, AKI, RM, normal acid-base balance, hypotension, shock, hyperosmolarity, ketonemia, ketonuria	Acute IHD in neurological symptoms and isopropranol levels > 200 mg/dL Supportive therapy
Organophosphates [111–113]	*Overstimulation of muscarinic ACR in PS*: miosis, hypotension, bradycardia, bronchial secretion, vomiting, salivation, urination, diarrhea, lacrimation *Overstimulation of nicotinic ACR in SyS and neuromuscular junction:* tachycardia, fasciculations, crumps, hypertension, sweating *Overstimulation ACR in CNS:* confusion, agitation, coma, RF	Atropine 1–3 mg IV bolus-double dose after 5 min if no effects Pralidoxime chloride 2 g IV over 20–30 min – infusion 0.5–1 g/h Supportive therapy – infusion of crystalloids, support of respiratory function Benzodiazepines IV in agitation HP, HDF, PF – in cases
Morphine, heroin, opiates [114,115]	Miosis, depressed mental status, decreased respiratory rate and bowel sounds, hypotension	Naloxone hydrochloride 0.2–1 mg initially can be repeated in 2–3 min intervals. in coma – 2 mg IV initially
Synthetic cannabinoids [116]	Hyperemesis, excessive loss of fluids, psychosis, agitation, convulsions, anxiety, RF, RM, AKI, dysrhythmias, cardiac arrest or cerebral ischemia	IV infusion of crystalloids, Antiemetics, Benzodiazepines and neuroleptics in psychosis or agitation Neurological and cardiological interventions in ischemic symptoms
Cocaine [96–98,117]	Agitation, psychosis, seizures, status epilepticus, hemorrhagic and ischemic stroke, hyperthermia, MI, DIC, RM, MODS, ACS, tachycardia, aortic dissection, hypertension, pneumomediastinum, pneumothorax, alveolar hemorrhage, bronchoconstriction, bowel ischemia and perforation, AKI – thrombosis of renal vessels, renal infarction, acute TIN, GN, thrombotic microangiopathy	Benzodiazepines IV in seizures, Calcium channel blockers, Labetalol, Nitroglycerin in hypertension or ACS Cardiological, surgical, angiological and neurological specific interventions in ischemic symptoms IV infusions of crystalloids 200 mL/h initially in RM and hyperthermia to achieve adequate urine output Cooling techniques (or Dantrolen) in hyperthermia
Amphetamines [102,118,119]	Hyperthermia, psychosis, paranoid form of schizophrenia, hepatotoxicity, DIC, hypertension, tachycardia, cardiomyopathy (Tako-tsubo), ACS, chest pain, arrhythmia, RM, AKI – ATN	Benzodiazepines e.g. Diazepam 5 mg IV bolus and repeat after 5–10 min if necessary Midazolam 2.5 mg IV bolus and repeat after 5–10 min if necessary Supportive care. Betablockers – labetalol, carvedilol, metoprolol, propranolol in hyperadrenergic syndrome
Heavy metals [120–123]	*Mercury* – hyperpigmentation, AKI-ATN, GN, nephrotic syndrome, loose teeth, bluish discoloration, nausea, vomiting, diarrhea, hematemesis, necrotic colitis, ataxia, tremor, dysartria *Lead***–** altered mental status, seizures, coma, abdominal colic, peripheral motor neuropathy, hypochromic microcytic anemia, fatigue, emotional lability, AKI or chronic TIN, decreased fertility, hypertension *Cadmium* – femoral bone pain, skeletal deformities, AKI, chronic nephropathy, proteinuria (Itai-itai disease) reduced steroidogenesis, hypertension, hyperkeratosis, acanthosis *Uranium***–** natural – high radioactivity – acute radiation syndrome *Depleted uranium* – AKI, chronic nephropathy, reduced bone growth, hepatotoxicity, emphysema, pulmonary fibrosis, lung cancer, affected cognitive function and locomotion, infectious and autoimmune diseases	*Chelators:* DTPA, DMSA, DMPS, d-penicillamine, dimercaprol, EDTA, EHBP, Siderophores such as catechoylamide and hydroxypyridones Calixarenes for skin decontamination Supportive care: gastric tube, GIT decontamination, activated charcoal administration 25 g every 4 h Additive therapy- Zn, Mg, Se, vitamins A, C, E, Natrium bicarbonate, hydrogen sulfide Plasma exchange in emergency situation (in cadmium poisoning) *Prevention*: Industrial activities: natural (*Moringa oleifera*) and chemical water decontamination
Paraquat (1,1-dimethyl-4,4-bipyridinium dichloride) [124–126]	Nausea, vomiting, gastric pain, mucosal lesions of oral cavity and pharynx, loss of consciousness, fever. Leukocytosis, anemia, acid-base disorders, acute hepatitis, AKI, RF, shock, hypotension	Supportive care, gastrointestinal decontamination, activated charcoal – 50 g in water into gastric tube Exposed skin wash with soap and water for up to 15 min HP – within 4 h of ingestion HD, HDF – in AKI Dexamethasone 8 mg IV every 8 h for 5 weeks Fluid resuscitation
Plants containing belladonna alkaloids (atropine effects) e.g. *Datura, Atropa belladona* [127,128]	Hallucinations (due to L-atropine, DL-hyoscyamine and hyoscine), loss of short term memory, agitation *Anticholinergic syndrome*: RF, cardiovascular collapse, dry skin, high fever, tachycardia, mydriasis, mucosal dryness, ileus, urinary retention – AKI, convulsion, coma	Physostigmine salicylate IV 0.5–2 mg slowly bolus, repeated according to clinical response after 20 min Supportive therapy, Gastrointestinal decontamination
Plants containing colchicine, e.g., *Gloriosa superba, Colchicinum autumnale* [128]	vomiting and severe diarrhea, dehydration, hemodynamic instability, hypotension, shock, AKI and eventually MODS	Anticolchicine antibodies (polyclonal Fab fragments) – no commercial production Supportive care fluid resuscitation IHD in AKI
Snake bites [62]	AKI, RM, DIC, shock, hypotension, local necrotic tissue, hemolysis	Specific antivenom serum Supportive therapy – fluid resuscitation

ACR: acetylcholine receptors; ACS: acute coronary syndrome; AKI: acute kidney injury; ARDS: acute respiratory distress syndrome; ATN: acute tubular necrosis; CNS: central nervous system; DIC: disseminated intravascular coagulation; DMSA: meso-2,3-dimercaptosuccinic acid; DMPS: 2,3-dimercapto-1-propanesulfonic acid; DTPA: diethylenetriamine pentaacetic acid; EDTA: ethylenediaminetetracetic acid; EG: ethylene glycol; EHBP: Ethane-1-hydroxy-1,1-bisphosphonate; GN: glomerulonephritis; HDF: hemodiafiltration; HP: hemoperfusion; IHD: intermittent hemodialysis; IV: intravenously; LD: loading dose; MAC: metabolic acidosis; MI: myocardial infarction; MODS: multiple organ dysfunction syndrome; PF: plasmapheresis; PS: parasympathetic system; RF: respiratory failure; RM: rhabdomyolysis; SyS: sympathetic system; TIN: tubulointerstitial nephritis.

## Diagnosis and management of selected intoxications and AKI

As mentioned, the diagnosis of AKI is generally based on measurement of: (1) increase in serum creatinine and (2) decreased urine output. Patients are divided into three groups according to the KDIGO recommendation for the classification of disease severity [1]. Both markers are the surrogates for decrease in glomerular filtration rate following renal damage after toxic insult.

Specific biomarkers for toxic AKI are not clearly established and some common biomarkers reflecting acute tubular cell injury have been studied with different results: N-acetyl-β-d-glucosaminidase (NAG), β_2_-microglobulin, Cystatin C, Interleukin-18 (IL-18), Kidney Injury Molecule-1 (KIM-1), Neutrophil gelatinase-associated lipocalin (NGAL) and Liver-type fatty acid binding protein (L-FABP). None of these biomarkers revealed any specificity for any one poisonous substance. A positive result can reflect tubular cell injury of any etiology. Moreover, many clinical conditions and parameters have influence on serum and urine concentration [61]. The other limitations of such biomarkers are based on their intrinsic preclusion for exact AKI diagnosis and prognosis and only as a part of a, panel of biomarkers [129].

There is current interest in microRNAs in the diagnosis of AKI due to a variety of etiological insults in critically ill patients. MicroRNAs are endogenous single-stranded short noncoding mRNAs of 19–23 nucleotides that repress and thus regulate the expression of almost 50% of known protein-coding genes with a regulatory role in modulating cellular processes such as the cell cycle, proliferation and type of death with influence on the whole pathophysiological outcome [130]. Many microRNAs have been identified as having a potential diagnostic role and therapeutic target in AKI. Some, such as miRNA-21 can have a double function with protection against renal injury by inhibiting apoptosis and inflammation, but also exacerbation of the injury response and promotion of fibrosis. Animal and experimental studies have been carried out to identify a microRNA implicated in toxic AKI namely in cisplatin or gentamicin nephropathy [131–133]. However, the considerable heterogeneity of published studies limits any immediate use of microRNAs as diagnostic or therapeutic targets in clinical applications. More comprehensive scientific research will be needed in the future to develop and explain the molecular role of microRNAs with targeted genes and transduction pathways involved [134].

The preventive and therapeutic approaches in critically ill patients with toxic AKI are: (1) specific for underlying diagnosis and (2) general – common for any patient with acute renal failure on ICU.

Strategies to minimize plant and medication-induced nephrotoxicity include quality control and standardization of herbal/drug products, research on the pathophysiological mechanisms of toxicity and adequate clinical trials to demonstrate efficacy and safety [56]. In the case of very nephrotoxic antibiotics such as vancomycin and aminoglycosides, the physician must use therapeutic drug monitoring to prevent nephrotoxic serum concentration and development of AKI.

The more specific and mostly international problem is adequate preventive measures as control of pesticide use. Related to these circumstances, the World Health Organization (WHO) with international financial support, introduced a project planning to reduce health risks through sound management of pesticides in 2013. The main targets of the project are: creating an evidence-based global pesticide registration and management practice in endemic countries, enhancing awareness and political support, developing new peer-reviewed guidelines for pesticide registration and management as well as providing technical support and training to priority countries. In addition to the above, each country should provide regulatory and quality control, disposal of waste, adequate legislation and strict control of pesticide poisoning or exposure [135].

Illicit drugs toxicity is a worldwide problem and needs the cooperation of several organizations including the WHO and international police forces. Prevention is based on developing national polices and action plans to reduce illicit drug demand and the incidence of associated infectious diseases such as hepatitis B, hepatitis C and HIV (WHO/Europe) [136]. Important is also the stopping of production and export of drugs mostly from developing countries. Furthermore, in recent years there is a big problem with internet drug shopping, especially in the case of synthetic cannabinoids which cannot be raced.

Therapeutic measures in the case of acute intoxication include appropriate antidote administration if possible in specific intoxication as a priority and general therapeutic approaches in poisoned patients.

General recommendation for prevention and treatment of critically ill patients with AKI are based on: (1) correction of hypovolemia using isotonic crystalloids with avoidance of hyperhydration, (2) regular monitoring of ions (chloride) and acid base-balance with adequate correction when chloride rich solutions are used, (3) use of vasopressor – norepinephrine to maintain mean arterial pressure 65–70 mmHg, (4) use of diuretic agent to avoid fluid overload and preserve diuresis if needed, (5) tight control of serum glucose, (6) therapeutic drug monitoring in initiation of therapy with highly nephrotoxic antimicrobials (e.g., vancomycin, gentamicin), (7) initiation of renal replacement therapy (RRT) according to the clinical picture or laboratory findings, usually in stage 2 or 3 according to KDIGO [1,137]. Renal replacement therapy can be used in two forms according to the duration and intensity needed: intermittent or continuous hemodialysis, hemofiltration or hemodiafiltration. Patient hemodynamic instability is usually a crucial factor for initiating continuous RRT.

Some toxic substances can be dialyzed or successfully removed from the circulation using a specific type of extracorporeal blood purification therapy (Table 1).

The clinical and biochemical criteria to initiate the RRT depend on many variables including the toxic substance (dialyzable or not), metabolic disorders and severity of renal impairment. Typically dialyzable are toxic alcohols such as ethylene glycol, methanol and ethanol. From clinical criteria for RRT initiation the most important are, hyperhydration without response to diuretics, anuria or diuresis <200 mL for at least 6–12 h and uremic symptoms with encephalopathy, lethargy, vomiting and diarrhea. Rapidly rising or life-threatening biochemical results indicate RRT in situations with hyperkalemia (K^+^ > 6.5 mmol/L), severe metabolic acidosis (pH < 7.1), or hyperazotemia depending on time of elevation [138]. Rhabdomyolysis is usually accompanied by some degree of AKI and high-flux and high-permeability membranes can be used in this situation [139].

This aside, specialized types of blood purification therapy can be used in patients with toxic AKI such as hemoperfusion on adsorbent columns when the toxin is lipophilic and high molecular weight. The charcoal-based sorbent, synthetic or anion exchange resins are available for the treatment of specific poisonings. The molecular adsorbent recirculating system is a type of albumin dialysis which can be used in patients intoxicated with *Amanita phalloides* and hepatorenal failure [140]. For physicians on general ICUs, it is essential to remember, blood purification and renal replacement therapy are important life-saving procedures with emphasis on specialized clinical staff and wide expertise in intensive care nephrology.

## Conclusions

Better knowledge of nephrotoxic substances leading to AKI will improve clinical practice in all fields of medicine. Historical information of the effects of many herbal substances is not sufficient for their routine use in current medicine. Many are still awaiting approval for use in pharmacology. Recovery of renal function without the need for chronic dialysis and patient survival are the basic criteria determining the use of different diagnostic and therapeutic approaches in critically ill patients. In the future, more research on the pathophysiological mechanisms of the toxicity of herbs and medical substances and adequate clinical trials will need to demonstrate efficacy and safety. Use of pesticides and other agrichemicals need adequate legislation, international registration of toxicity and more strict control. Illicit drugs addiction is a big international problem, which need to be solved by more organizations including WHO.
